# Exploring the intersection of brain injury and mental health in survivors of intimate partner violence: A scoping review

**DOI:** 10.3389/fpubh.2023.1100549

**Published:** 2023-03-02

**Authors:** Danielle Toccalino, Amy Moore, Elizabeth Cripps, Sophia Chuon Gutierrez, Angela Colantonio, Christine M. Wickens, Vincy Chan, Emily Nalder, Halina (Lin) Haag

**Affiliations:** ^1^Institute of Health Policy, Management and Evaluation, University of Toronto, Toronto, ON, Canada; ^2^Acquired Brain Injury Research Lab, University of Toronto, Toronto, ON, Canada; ^3^Lyle S. Hallman Faculty of Social Work, Wilfrid Laurier University, Waterloo, ON, Canada; ^4^Faculty of Kinesiology and Physical Education, University of Toronto, Toronto, ON, Canada; ^5^KITE-Toronto Rehabilitation Institute, University Health Network, Toronto, ON, Canada; ^6^Department of Occupational Science and Occupational Therapy, Temerty Faculty of Medicine, University of Toronto, Toronto, ON, Canada; ^7^Rehabilitation Sciences Institute, Temerty Faculty of Medicine, University of Toronto, Toronto, ON, Canada; ^8^Dalla Lana School of Public Health, University of Toronto, Toronto, ON, Canada; ^9^Institute for Mental Health Policy Research, Centre for Addiction and Mental Health, Toronto, ON, Canada; ^10^Campbell Family Mental Health Research Institute, Centre for Addiction and Mental Health, Toronto, ON, Canada; ^11^Department of Pharmacology and Toxicology, University of Toronto, Toronto, ON, Canada

**Keywords:** intimate partner violence (IPV), brain injury—traumatic, brain injury, strangulation, mental health, health services research

## Abstract

**Rationale:**

Intimate partner violence (IPV) is the most commonly occurring form of violence against women. The most common site of injury in IPV is the head, face, and neck, resulting in possible brain injury (BI). Independently, mental health (MH) concerns are highly prevalent among both IPV survivors and individuals with BI; however, no systematic review exists on the combined experience of BI and MH in IPV.

**Objective:**

The aim of this review was to describe the identification of and relationships between BI, MH, and IPV in the literature and the implications for health policy and practice.

**Methods:**

A search strategy including text words and subject headings related to BI, IPV, and MH was developed for MEDLINE and translated to EMBASE, PsycINFO, CINAHL, Cochrane, Scopus, and Web of Science. Two reviewers independently assessed articles for inclusion. Articles discussing MH, BI, and IPV in relation to one another were included in the review.

**Results:**

Twenty-eight articles were identified for inclusion. Methods for identifying IPV, BI, and MH were highly variable across studies. Fourteen studies reported significantly higher MH scores in IPV survivors with BI than in those without BI. Articles predominantly focused on cis gender women in heterosexual relationships and the impact of race and ethnicity were largely overlooked. Healthcare access was explored by eight articles, though none discussed the implications of co-occurring BI and MH.

**Conclusion:**

Brain injury and MH are highly prevalent among IPV survivors; however, little research discusses the implication for healthcare. Future research should explore healthcare-related needs and experiences to inform policy and practice and better represent the diversity of IPV survivors.

## 1. Introduction

Recent estimates suggest 44% of women and 36% of men will experience intimate partner violence (IPV) in their lifetime, more than half of whom will experience physical violence (1). Intimate partner violence has been defined as physical, psychological, or sexual violence committed by an intimate partner or ex-partner and can result in significant emotional and bodily harm (2). Individuals of all genders and sexual orientations experience IPV; however, most research has focused on women survivors of IPV. Women experience higher rates and more severe forms of IPV than men, including higher rates of strangulation (1), and IPV is the most commonly experienced form of violence women experience (3, 4). For the purposes of this review, we also include individuals working in sex work or prostitution under the umbrella of IPV. An estimated 45–81% of sex workers experience violence from their clients and many also experience violence from another intimate partner (5, 6).

The COVID-19 pandemic has exacerbated IPV globally, significantly increasing both rates of IPV and the level of violence per encounter (7–11). Physical violence in IPV most commonly results in injury to the head, face, and neck (12), leaving survivors at high risk of traumatic brain injury (TBI). TBI is “an injury to the brain producing an alteration in brain function, or other evidence of brain pathology, caused by an external force” (13). Strangulation, also commonly experienced during IPV (1, 14), can result in hypoxic-ischemic brain injury due to a lack of blood circulation and consequently oxygen and nutrients to the brain (15, 16). Both hypoxic-ischemic and traumatic brain injuries have been included in this review under the umbrella of brain injury (BI), as the context of IPV similarly informs treatment and recovery challenges for both injuries (17–19).

Brain injuries from any cause can have significant long-term cognitive, psychiatric, physical, and social consequences (20–23). Previous research indicates a high prevalence of BI among IPV survivors (14, 24), suggesting a significant need for more attention to IPV-related BI. However, lack of awareness, gaps in screening, and unique challenges in healthcare access often leave BI overlooked in IPV survivors, hindering identification and support (14, 17). Identification of IPV-related BI is further challenged by the high correlation between symptoms and sequelae of BI and symptoms of mental health (MH) concerns that are also commonly experienced by survivors (25–31). A recent Lancet Psychiatry Commission report focused on the intersection of IPV and MH noted the elevated risk of MH concerns among IPV survivors and the heightened risk of IPV among individuals, specifically women, with MH concerns (32). However, the report made no mention of head injury or BI of any kind, which is suggestive of the work still to be done in recognizing the triple intersection of IPV, MH concerns, and BI. The correlation between BI symptoms and MH concerns makes differential diagnosis difficult and further complicates the provision of and access to adequate and appropriate healthcare (25, 33). The interaction between BI and MH concerns can impact care and treatment for the BI, MH concern, or both. For example, a BI can amplify the symptoms of post-traumatic stress disorder (PTSD), anxiety, or depression, making these MH concerns more difficult to treat if the underlying BI goes unaddressed (27, 33). Furthermore, treatment for MH concerns may be more effective when accommodations are made for potential difficulties with emotion regulation, impulse control, pain, and cognitive limitations that can accompany BI (30).

Despite the high rates of both BI and MH concerns among survivors of IPV and the high rates of MH concerns among individuals with BI, the literature investigating the co-occurrence and combined experience of BI and MH concerns among survivors of IPV (triple intersection) is limited, and there has not yet, to our knowledge, been a comprehensive review investigating the intersection of BI and MH concerns among IPV survivors. An investigation into the triple intersection is needed as most of the research currently informing BI guidelines is based on predominantly male samples injured through other mechanisms (e.g., sports, military service).

This scoping review was developed to explore what is known in the literature about MH concerns and BI among survivors of IPV. Specifically, it aimed to summarize and synthesize the existing literature through the following objectives: (1) describe how IPV, BI, and MH concerns are identified in the literature and (2) describe the relationships between IPV, BI, and MH concerns. BI is often overlooked in IPV survivors with significant health implications, and MH concerns further complicate healthcare provision and access. Therefore, a third objective was to identify the implications for healthcare and health systems to inform policy and practice.

## 2. Methods

This scoping review looked at MH concerns and BI among survivors of IPV as reported in the published literature since the inception of the searched databases. The review was designed following the framework first developed by Arksey and O'Malley (34) and further developed by JBI, formerly the Joanna Briggs Institute (35, 36). Reporting was guided by the Preferred Reporting Items for Systematic reviews and Meta-Analyses extension for Scoping Reviews (PRISMA-ScR) Checklist (37). The search strategy and eligibility criteria were informed by a previous scoping review investigating BI among survivors of IPV (14). Search terms for MH concerns were informed by a previous systematic review investigating MH and BI (38, 39) and by the literature exploring MH implications of both BI and IPV (14, 25–28, 40–43).

### 2.1. Search strategy

MEDLINE, EMBASE, PsycINFO, CINAHL, Cochrane, Scopus, and Web of Science were searched for relevant articles using a search strategy including text words and subject headings (e.g., MeSH, Emtree) related to BI, IPV, and MH. The search was initially run in October 2020 and revised and updated in January 2022 using concepts broadly characterized as follows:

1. **Brain injury:** Traumatic brain injury, concussion, head injury, post-concussion syndrome, strangulation, choking, face injury, and neck injury.
2. **Intimate partner violence:** Domestic violence, spousal abuse, spouse abuse, intimate partner violence, interpersonal violence, battered women, intimate violence, and sex work.
3. **Mental health:** Post-traumatic stress disorder, anxiety, depression, bipolar and related disorders, mood disorders, obsessive-compulsive disorder, phobias, substance use disorder, drug abuse, and alcohol abuse.

Sex workers are often excluded from IPV; however, there are many similarities in the violent encounters experienced by sex workers and IPV survivors. As such, previous reviews have chosen to include both IPV and sex work terms in their search (14), which we mirror in this review. For the complete search strategy, refer to Appendix A.

Searches were not limited by language, year of publication, or geographic location. Returned records were managed in EndNote and Covidence (44). A manual search of the reference lists of each article meeting the full-text inclusion criteria as well as any identified review articles discussing BI, MH concerns, and IPV was conducted to identify additional literature not captured in the original search.

### 2.2. Eligibility criteria: Title and abstract screen

Following the removal of duplicates, two reviewers (DT and either AM, SCG, or EC) independently assessed all identified titles and abstracts for eligibility. This screen focused on identifying primary research studies, including theses or dissertations, and review articles addressing BI among IPV survivors, MH concerns among IPV survivors, or IPV among individuals with BI. This broad approach was taken based on previous experience with reviews on BI suggesting that all relevant subgroups (in this case, IPV, MH, and BI) are not always included in the abstract, though relevant data may be presented in the body of the article. Articles were excluded if they focused on the perpetrator, on populations younger than 18 years of age, or on violence outside of the context of an intimate partner relationship. Conference abstracts, protocols, books or book reviews, and animal studies were also excluded. Covidence software was used for screening and to monitor agreement between the reviewers' assessments (87–94% agreement between pairs). Differences were resolved through discussion and consensus; articles were moved to the full-text screen if consensus could not be reached.

### 2.3. Eligibility criteria: Full-text screen

Full texts were again reviewed independently by two reviewers (DT and either AM, SCG, or EC). For inclusion in the review, studies needed to specifically discuss MH concerns and BI in survivors of IPV, be written in English, and be available through the University of Toronto Library system. Exclusion criteria used for the abstract and title screen continued to apply. In addition, articles were excluded if they were commentaries or if they did not discuss MH, BI, and IPV in relation to one another (e.g., discussing MH and BI separately). As with the title and abstract screen, Covidence software was used to conduct the screening and monitor agreement between the reviewers' assessments (80–96% agreement between pairs). All differences in screening were resolved through discussion and consensus.

### 2.4. Data extraction and synthesis

Study details (i.e., location, design, population, sample size, data source, definitions for IPV, BI, and MH) and key findings (prevalence of IPV, BI, and MH; healthcare use; relationships between IPV, BI, and MH) were extracted from included studies as reported. Data were extracted by one reviewer and peer-reviewed by a second (DT and AM or EC) then synthesized using narrative synthesis (45).

## 3. Results

**Figure 1:**
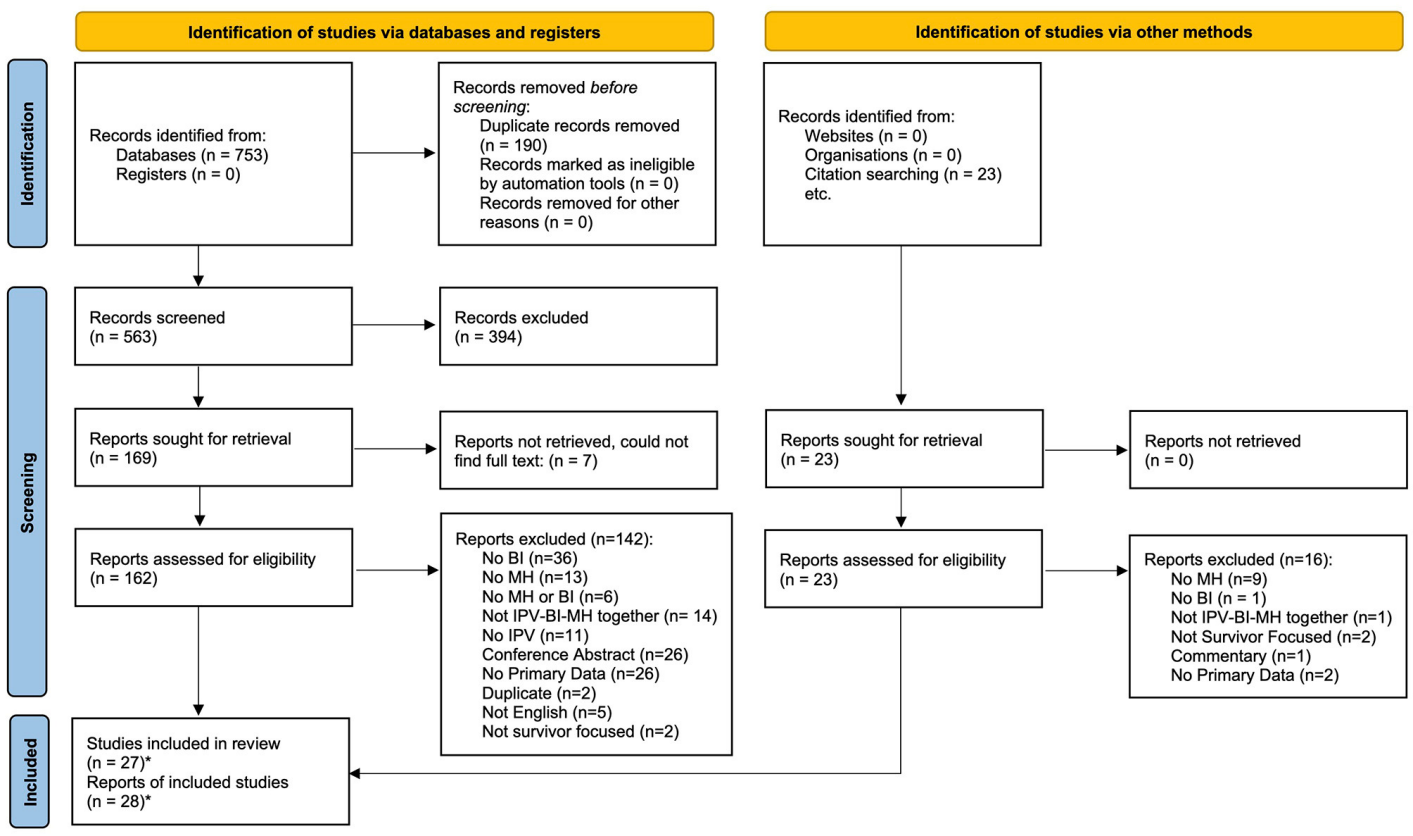
PRISMA 2020 flow diagram for new systematic reviews which included searches of databases, registers and other sources. *Two of the included articles reported on the same study, resulting in 28 total articles being included reporting on the findings from 27 studies. From Page et al. (46).

Searching the seven databases returned 753 results and 563 unique records following duplicate removal. From this body of literature, a total of 28 articles reporting on 27 studies (including three theses) were included. For a comprehensive breakdown of the article review process, please refer to Figure 1.

### 3.1. Article characteristics and study populations

Articles were predominantly published in the last 5 years (61%, *n* = 17) and based on data from the United States (US; 82%, *n* = 23). Study populations consisted almost exclusively of women or female survivors, with a few noted exceptions. Three studies included male survivors in their sample, accounting for 3–10% of the study populations (47–49). Gabbe et al. (50) found 27% of major trauma patients presenting with TBI caused by IPV-related violence were male. While both male and female survivors were included in these studies, sex- or gender-specific findings were not reported, though small sample sizes were likely prohibitive of that reporting. Four studies specifically reported on the sex or gender of the perpetrator, all specifying males or men as perpetrators (51–54). One additional study noted the study population as heterosexual women (55).

Two studies specifically explored the experiences of Black or African American women (56, 57), and one study focused on the experiences of Chinese women (58). The remainder of the studies had variable reporting on race or ethnicity. Four articles reported on the percentage of participants that were non-white (ranging from 4 to 62% stratified by BI status) (27, 59, 60) or from a visible minority (13%) (61). Five articles reported on the number of participants who were Black/African American or white with the remainder in a mixed race or other categories (40, 52, 55, 62, 63). Nine articles reported on all groups represented in the sample (10, 43, 47, 49, 51, 54, 64–66), and seven articles did not report race or ethnicity at all (48, 50, 53, 67–70). Among studies where race or ethnicity was not an inclusion criterion, white (*n* = 14 studies, 8–75%), African American/Black (*n* = 13, 13–90%), and Latina/Hispanic (*n* = 6, 1–16%) were the most commonly reported groups. Some studies controlled for sociodemographic factors (including race or ethnicity) in their analyses; however, none reported race- or ethnicity-specific findings.

Though the search terms were broadened to include sex work, none of the included studies focused on or included individuals who participate in sex work or prostitution. However, the inclusion of strangulation in our search terms was mirrored to a large extent in the literature. Although most articles referred to TBI, 15 of the 28 included articles (54%) included strangulation in their definition of TBI. In addition, five articles focused specifically on strangulation (51, 54, 55, 66, 69). One of these articles, reporting on findings from a broader study investigating BI (43), specifically looked at strangulation-related alterations in consciousness (66); however, three of the remaining four articles reported high rates of loss of consciousness or dizziness among their study participants, indicative of a potential BI (51, 54, 69). For article summaries, refer to Table 1.

**Table 1:** Summary of included articles.

**Author [year] Country**	**Objective and study design**	**Study setting/population [sample size]**	**Identification of IPV**	**Assessment of BI**	**Assessment of MH**	**IPV**	**BI (among IPV survivors)**	**MH (among survivors w/BI or stratified by BI status)**
Joshi et al. (2012) (51), United States	**Objective:** Explore women's perceptions and experiences of intimate partner strangulation. **Design:** Exploratory study using qualitative interviews and focus groups	Adult women experiencing physical abuse by a male partner in the last year [*N* = 17]	**Past year IPV:** Recruitment through a domestic abuse shelter ^*^Inclusion criterion	**Strangulation** “In the last 12 months, has an intimate partner ever tried to physically assault you by choking you, or putting his hands around your throat and squeezing it, or putting a piece of clothing/ wire/ cord around your throat and pulling it tightly?”	**General:** Self-report during interviews and focus groups	100%	**Strangulation:** 100% **LOC:** 82% (*n* = 14) 11.8% (*n* = 2) reported being ‘close to blacking out'	Psychological problems including nightmares, insomnia, anxiety, and heightened and persistent fear. Some women reported that existing problems such as depression, anxiety, and suicidal ideation worsened after strangulation. Women's mental health problems regularly continued beyond the abusive relationship into a new intimate relationship.
Smith et al. (2001) (69), United States	**Objective:** Determine if there is a correlation between the number of times a victim of IPV has been manually strangled and the frequency of symptom development during the 2 weeks following the attack(s). **Design:** Cross-sectional, observational	Female respondents recruited from the Parkland Health and Hospital System, Violence Intervention and Prevention Center, and Emergency Department in Dallas and domestic violence shelters in Dallas/Fort Worth, Texas and Los Angeles, California. [*N* = 101]	**Lifetime IPV:** Self report: Current or previous involvement in an abusive relationship ^*^Inclusion criterion	**Strangulation** Self report: survey responses from female subjects reporting a history of strangulation	**General:** Self report of specific medical symptoms related to the physical and mental health collected *via* survey	100%	**Strangulation:** 100% Single strangulation event: 44% (*n* = 44) 2–5 strangulation events: 34% (*n* = 34) More than 5 strangulation events: 23% (*n* = 23)	>50% of single attack victims reported the development of symptoms related to psychological health reported in five of the seven survey inquiries (personality changes, depression, nightmares, insomnia, suicidal ideation, anxiety, PTSD diagnosis). Significant increases in the frequency of nightmares reported among females with >5 strangulation events.
Wilbur et al. (2001) (54), United States	**Objective:** Determine the incidence of strangulation within the cycle of violence; and examine subjective medical symptoms experienced, and elective utilization of health care by female victims of non-lethal intimate partner strangulation. **Design:** Cross-sectional survey	The Parkland Health and Hospital System, Violence Intervention and Prevention Center, a domestic violence women's shelter in inner city Dallas, and a women's shelter in inner city Los Angeles [*N* = 62]	**Lifetime IPV:** Self report: Current or previous involvement in an abusive relationship ^*^Inclusion criterion	**Strangulation** “Have you ever been strangled”	**General:** Medical symptoms assessed *via* survey	100%	**Strangulation:** 68% (*n* = 42) Intimate Partner: 61% (*n* = 38) Friend or family member: 6% (*n* = 4) 3+ times: 43% (*n* = 19/41) **Dizziness:** 61% (*n* = 25/41) **LOC:** 17% (*n* = 7/41)	**Depression:** 81% (*n* = 30/37) **Suicidal ideation:** 31% (12/39) **Anxiety:** 83% (*n* = 33/40)
Mittal et al. (2018) (55), United States	**Objective:** Examine the associations between strangulation and depressive symptoms among a sample of help-seeking women reporting IPV. **Design:** Cross-sectional, secondary data analysis from RCT (HIV-IPV prevention)	Heterosexual women reporting IPV recruited through DV agencies, public health clinics, MH agencies, substance abuse clinics, and hospitals [*N* = 175]	**IPV in last 3 months:** Abuse Behavior Inventory and WEB ^*^Experience of abuse in last 3 months an inclusion criterion	**Strangulation** 5-point scale ranging from 0 (never) to 4 (very frequently), if their partner had strangled them in the last 3 months.	**Depression**: CESD 9-item scale	100% Significantly higher WEB and Abuse Behavior Inventory scores among women who were strangled (*p* ≤ 0.01)	**Strangulation:** 59% (*n* = 103)	**Depression (mean score** **±** **SD):** Total (*N* = 175): 4.32 ± 2.95; Strangulation (*n* = 103): 4.88 ± 2.76; No strangulation (*n* = 72): 3.58 ± 3.08; Significant difference (*p* < 0.01)
^§^Valera et al. (2022) (66), United States	**Objective:** Explore the relationship between strangulation-related alterations in consciousness and cognitive and psychological outcomes independent of TBIs. **Design:** Cross-sectional, retrospective	Women recruited from shelters, programs for relationship support, protection order assistance, substance abuse support, and snowball sampling. [Rate of strangulation in IPV: *N* = 99, All other analyses: *n* = 52] ^*^47 excluded from further analysis due to prior conditions	**Lifetime IPV:** Self report ^*^Physical IPV an inclusion criterion	**Strangulation** BISA was used to assess a history of IPV- and non-IPV-related alterations in consciousness, including those related to strangulation and TBIs. ^*^Moderate to severe TBI (IPV-related or other) and mTBI from accidents in the past 12 months excluded for analyses	**PTSD:** CAPS-2 **Anxiety and depression:** The Mood and Anxiety Symptom Questionnaire—Short Form ^*^Drug or alcohol dependence in last 6 months, bipolar disorder, and schizophrenia excluded	100%	**Strangulation-related alteration in consciousness:** 27% (*n* = 26/99); 29% (*n* = 15/52)	**Anhedonic Depression (mean score** **±** **SD):** Total: *N* = 52; Strangulation: *n* = 15; No strangulation: *n* = 37. Total: 63.1 ± 16.1; Strangulation: 70.9 ± 12.5; No strangulation: 60.1 ± 16.5; Significant difference (*p* = 0.03). **PTSD (mean score** **±** **SD):** Total: 18.4 ± 24.2; Strangulation: 31.4 ± 30.5; No strangulation: 13.2 ± 19.3; Significant difference (*p* = 0.02). **Anxious arousal (mean score** **±** **SD):** Total: 26.6 ± 8.1; Strangulation: 27.4 ± 7.9; No strangulation: 26.3 ± 8.3; No significant difference (*p* = 0.67)
Iverson et al. (2019) (27), United States	**Objective:** Examine the psychosocial health risks associated with IPV-related TBI history in women veterans. **Design:** Longitudinal, survey-based study	Women veterans participating in the KnowledgePanel who reported TBI at time 1 and participated in the time 2 survey [*N* = 33]	**Lifetime IPV:** modified HARK tool. ^*^IPV an inclusion criterion	**TBI and Strangulation** VA TBI screening tool. Assessed in two stages: (1) experienced an IPV-related head event (incl. strangulation) and (2) had altered or loss of consciousness following the IPV-related head event. ^*^BI at time 1 an inclusion criterion	**PTSD:** PCL-5 **Depression:** CESD-10 **Anxiety:** DASS.	100%	**IPV-Related BI:** 100% w/persistent symptoms: 39% (*n* = 13) w/o persistent symptoms: 61% (*n* = 20) Persistent symptoms began or got worse following IPV-related head event and occurred in the past week.	**PTSD (mean score** **±** **SD)**: 23.18 ± 21.65 **Depression (mean score** **±** **SD)**: 20.36 ± 13.67 **Anxiety (mean score** **±** **SD)**: 8.91 ± 9.67 IPV-related TBI w/ persistent symptoms at Time 1 was associated w/ significantly worse outcomes at Time 2 across all health outcome domains. Controlling for PTSD, IPV-related TBI w/ persistent symptoms at Time 1 remained significantly associated w/ worse Time 2 symptoms of insomnia, depression, and physical health.
Ralston et al. (2019) (48), United States	**Objective:** Identify the signs and symptoms frequently presenting in IPV on a forensic nursing exam that are consistent with TBI. **Design:** Retrospective forensic nursing report review	Patients (17 female, 2 male) presenting as victims of strangulation to a family advocacy center. [*N* = 19]	**Recent IPV:** Presenting to the family advocacy center as a victim of IPV ^*^Inclusion criterion	**TBI and Strangulation:** Presenting as a victim of strangulation, reporting mechanisms of injury to the head. ^*^Strangulation an inclusion criterion	Self-reported past medical history	100%	**Strangulation:** 100%. **Blow to the Head:** 68.4% (*n* = 13) (*via* hand or object) either before (*n* = 8) or after (*n* = 5) being strangled	42.1% (*n* = 8) had at least one documented mental illness (not otherwise specified)
Saadi et al. (2021) (53), United States	**Objective:** Discuss the overall approach to the forensic evaluation of asylum-seekers w/ history of TBI **Design:** Case Vignettes	Assylum seekers with BI (2 referred to using she/her pronouns, 1 using he/him pronouns) to the US [*N* = 3]	Self-report	**TBI and Strangulation** Self-reports of hits to the head or ‘choking'	**PTSD:** Breslau's 7-item screen (cutoff of >4) Harvard Trauma Questionnaire (cutoff of >2.5)	66.6% (*n* = 2, both women)	**Head Injury:** Both IPV survivors reported at least one head injury with LOC. One reported strangulation without LOC.	One IPV survivor assessed and screened positive for PTSD on the Breslau's 7-item screen the Harvard Trauma Questionnaire
Cimino et al. (2019) (56), United States	**Objective:** Examine the relationship between IPV, injuries associated with TBI, and their effect on mental health disorders among abused Black women. **Design:** Secondary analysis of a retrospective cohort study	Black or African American abused women participating in the ESSENCE Project recruited from two Baltimore City public health STD clinics [*N* = 95]	**Lifetime IPV:** CTS-2	**TBI and Strangulation** LOC from a blow to the head (CTS-2) and/or from being choked (study screening survey ‘has your partner ever choked you until you became unconscious?')	**Depression:** CESD-10 **PTSD:** NSESSS	100%	**Probable TBI:** 33.7% (*n* = 32) **LOC from blow to head:** *n* = 12 **LOC from strangulation:** *n* = 12 **Strangled and hit on the head in the last year:** *n* = 8 **Multiple LOC:** *n* = 9	**Depression (mean score** **±** **SD)** Probable TBI: *n* = 32; No probable TBI: *n* = 63 Probable TBI: 15.3 ± 7.09 No Probable TBI (*n* = 63): 12.6 ± 6.95 Not statistically significant **PTSD (mean score** **±** **SD)** Probable TBI (*n* = 32): 24.8 ± 8.08 No Probable TBI (*n* = 63): 16.9 ± 9.95 Statistically significant (p < 0.001) **Comorbid scores (mean score** **±** **SD)** Probable TBI (*n* = 32): 40.1 ± 13.3 No Probable TBI (*n* = 63): 29.5 ± 14.8 Statistically significant (*p* < 0.001).
Iverson et al. (2017) (27), United States	**Objective:** Identify the occurrence of IPV-related TBI and associated PTSD symptoms among women veterans who experienced IPV. **Design:** Cross-sectional survey	Women veterans participating in the KnowledgePanel who completed a follow up survey and had experienced IPV [*N* = 224]	**Past-year IPV:** HARK tool (4-item)	**TBI and Strangulation** VA TBI screening tool. (1) experienced an IPV-related head event, and (2) had altered or loss of consciousness following the IPV-related head event. Persistent symptoms began or got worse following IPV-related head event and occurred in the past week.	**PTSD:** PCL-5 A cut-off score of 33 was used to determine probable PTSD diagnosis (yes/no).	100%	**IPV-related TBI:** 28% (*n* = 63)	**Probable PTSD Diagnosis (PCL-5** **≥** **33)** IPV-related TBI w/ current symptoms (*n* = 28): 64.3% (*n* = 18) IPV-related TBI w/o current symptoms (*n* = 35): 29.4% (*n* = 10) No IPV-related TBI (*n* = 161): 16.9% (*n* = 27) When adjusting for race, income, and past-year IPV, women with IPV-related TBI w/ current symptoms were 5.9 times more likely to have probable IPV-related PTSD than those w/ no IPV-related TBI history.
Iverson and Pogoda (2015) (60), United States	**Objective:** Identify the occurrence of IPV-related TBI in women veterans and examine the associations of IPV-related TBI with sociodemographic characteristics, health symptoms, healthcare utilization, and IPV experiences. **Design:** Cross-sectional mail survey	New England Department of Veteran Affairs women veteran patients [*N* = 176]	**Past-year and lifetime IPV:** CTS-2	**TBI and Strangulation** VA TBI screening tool. (1) experienced an IPV-related head event, and (2) had altered or loss of consciousness following the IPV-related head event (IPV-related TBI).	**Depression:** CESD **PTSD:** PCL	100%	**IPV-related TBI:** 18.8% (*n* = 33)	**Depression (mean score** **±** **SD)** IPV-related head event **and** TBI (*n* = 33): 26.6 ± 9.7 IPV-related head event no TBI (*n* = 24): 20.7 ± 6.0 No IPV-related head event (*n* = 119): 18.8 ± 8.5 TBI group significantly different (*p* < 0.001) **PTSD (mean score** **±** **SD**) IPV-related head event **and** TBI (*n* = 33): 53.2 ± 17.2 IPV-related head event no TBI (*n* = 24): 34.1 ± 12.2 No IPV-related head event (*n* = 119): 32.9 ± 14.8 TBI group significantly different (*p* < 0.001)
^§^Valera and Berenbaum (2003) (43), United States	**Objective:** Examine if battered women are sustaining brain injuries from their partners and whether brain injuries are associated with partner abuse severity, cognitive functioning, or psychopathology. **Design:** Cross-sectional descriptive	Women who had sustained any physical abuse by a current or past intimate partner recruited from battered women's shelters and community program [Rate of IPV-TBI: *N* = 99, All other analyses: *n* = 57] ^*^42 excluded from cognitive functioning assessments due to prior conditions	**Lifetime IPV:** Severity measured by the CTS and Severity of Violence Against Women Scale ^*^Physical IPV an inclusion criterion	**TBI and Strangulation** Semistructured interview based on the Committee on Mild Traumatic Brain Injury's definition of mTBI ^*^Moderate to severe accident-related TBI (lifetime) and mTBI from accidents in the past 12 months excluded for analyses	**PTSD:** CAPS-2 **Depression and Anxiety:** Mood and Anxiety Symptom Questionnaire-Short Form **Susbstance use:** Psychoactive Substance Use module from Structured Clinical Interview for DSM-IV ^*^Drug or alcohol dependence in last 6 months, bipolar disorder, and schizophrenia excluded	100%	**IPV-related BI:** 74% Multiple: 51%. **Accident-related BI:** 27% Multiple: 5%	Not independently reported. After removing shared variance with partner abuse severity, small to moderate, statistically significant associations remained between the BI score and general distress, anhedonic depression, anxious arousal, and PTSD symptom severity. Greater levels of partner abuse severity are highly associated with higher levels of several of the psychopathology variables: anhedonic depression, worry, and PTSD symptom severity. However, these associations are attenuated and no longer statistically significant after removing shared variance with BI severity
Rajaram et al. (2021) (70), United States	**Objective:** Determine the extent of possible BI among IPV survivors to better understand the impact of these injuries **Design:** Cross-sectional descriptive	Women who had experienced IPV and accessed services at one of three community-based organizations (shelter, advocacy **and** social services) in the Midwest [*N* = 171]	**Lifetime IPV:** Experienced IPV and accessed services at IPV community-based organizations ^*^Inclusion criterion	**TBI and Strangulation** Modified HELPS tool assessing strangulations in addition to being hit in the head; when and how they had a hit to the head or were strangled; how many times they got hit in the head or were strangled.	**Anxiety and Depression: “**Problems” due to hit to the head or strangulation as measured with the HELPS tool	100%	**Positive HELPS:** 58% (*n* = 100); Higher proportions screened positive in the 20–40 (61%) and >40 (75%) age groups **Hit in the head/strangled:** 91% > 4 times: 52% LOC or Dazed/confused: 64%	**Experiences of participants ever hit in the head or strangled: Feeling anxiety:** *n* = 82 **Depression:** *n* = 81
Saleem et al. (2022) (49), United States	**Objective:** Identifiy the prevalence and risk factors of IPV-related ABI among the visitors to a New York Justice Center. Assess which symptoms are most associated with a positive mTBI screen. **Design:** Retrospective, cross-sectional study	Female (95%) and male (5%) IPV survivors recruited from a Justice Center providing resources to IPV survivors in New York. [*N* = 40]	**Last 60 Days IPV:** Self-report at least one incident of physical abuse in past 60 days. ^*^Inclusion criterion	**TBI and Strangulation:** Modified HELPS tool (both), Danger Assessment-Revised tool (strangulation). *Individuals with history of trauma to head/ face/ neck from non-IPV causes (e.g., motor vehicle accidents, disease) were excluded*	**Anxiety and Depression: “**Problems” due to hit to the head or strangulation as measured with the HELPS tool	100%	**Positive HELPS:** 40% (*n* = 16). **History of BI:** 100%; TBI: 100% (*n* = 40); Strangulation: 92.5% (*n* = 37); LOC: 42.5% (*n* = 17)	**Depression:** 25% (*n* = 10). **Anxiety:** 85% (*n* = 34). **History of Substance use:** 28.4% (*n* = 25)
Fortier et al. (2021) (62), United States	**Objective:** Validate the adapted BAT-L/IPV using the OSU-TBI-ID scoring method as the criterion standard for TBI diagnosis, and report the prevalence of head injury. **Design:** Semistructured clinical interview.	Women in greater St. Louis, Missourri who had experienced at least one IPV event in their lifetime and screened positive for probable PTSD. [*N* = 51]	**Lifetime IPV:** Lifetime trauma interview for IPV survivors ^*^Inclusion criterion	**TBI and Strangulation** BAT-L/IPV semistructured interview for TBI compared to OSU-TBI-ID **Post concussive symptoms:** Neurobehavioral Symptom Inventory (NSI)	**PTSD:** CAPS-5 ^*^Positive screen for probable PTSD on the PCL-5 an inclusion criterion	**Any IPV:** 100% **Physical IPV:** 96%	**Subconcussive IPV-head injury:** 76.5% **IPV-TBI:** 35.3% **Strangulation:** 31.4% (*n* = 16) LOC: 7.8% (*n* = 4) **BAT-L vs. OSU-TBI:** 43.4% (*n* = 50) of 115 head injury events were positive on both measures; *n* = 7 positive on one but not both **Subconcussive Non-IPV head injury:** 58.8% (*n* = 30) **Non-IPV-TBI:** 37.3% (*n* = 19)	**PTSD:** 100% screened positive (PCL-5) CAPS: 80.4% (*n* = 41), with the remainder (*n* = 10) subthreshold for PTSD PTSD severity (CAPS-5) 35.1 ± 7.1 PTSD severity (PCL-5) 48.7 ± 12.7
Smirl et al. (2019) (68), Canada	**Objective:** Examine the extent symptoms associated with potential TBI in IPV survivors overlap with sport-related concussions. **Design:** Exploratory pilot study, cross sectional design	Female IPV survivors recruited from partner sites [*N* = 18]	**Lifetime IPV:** Any woman who had experienced any form of abuse by an intimate partner ^*^Inclusion criterion	**TBI and Strangulation** BISA altered to include strangulation—assessed for any TBI, not just IPV-related; Sport concussion assessment tool 5th edition	**PTSD**: CAPS **Depression:** BDI **Anxiety:** BAI	100%	**TBI and Strangulation:** Average BISA score 4.3 ± 1.8 (range 1–8) indicating all participants had at least one previous TBI; **Strangulation:** 56%	**PTSD:** 94% reported symptoms consistent with varying degrees of PTSD **Anxiety and/or Depression:** 61% reported moderate to severe levels
Galovski et al. (2021) (63), United States	**Objective:** Evaluate massed cognitive processing therapy (CPT) delivered in an individual format over 5 days as compared with CPT delivered traditionally (i.e., weekly sessions) for women IPV survivors with PTSD. **Design:** Multiple subject, single case design of six matched pairs to compare treatment types and length	Females sex with lifetime experience of physical, sexual, and/or emotional IPV and a current PTSD diagnosis. [*N* = 12]	Clinician-administered interview ^*^IPV an inclusion criterion	**TBI and Strangulation** BAT-L/IPV version	**PTSD:** CAPS-5, PCL-5 **Psychiatric Comorbidity:** SCID-5 **Depression and Anxiety:** DASS-21, PHQ-9 ^*^Current PTSD Diagnosis an inclusion criterion	100%	**Head injury:** 92% (2.5 ± 2.15 events) **TBI diagnosis:** 41.7% (*n* = 5) **Strangulation by partner:** 41.7% (*n* = 5) [LOC in one]	Individuals w/ TBI had significantly higher PTSD severity at 1-month posttreatment compared to those without TBI (*p* < 0.001)
Maldonado-Rodriguez et al. (2021) (61), Canada	**Objective:** Characterize cognitive-motor function in women who have experienced IPV and examine the extent to which it was related to clinical measures of executive function **Design:** Exploratory pilot study, cross sectional design	Women who had experienced IPV were recruited from a local women's shelter and other women-serving organizations [*N* = 40]	WEB scale ^*^Inclusion criterion	**TBI and Strangulation** BISA	**PTSD:** CAPS-IV **Depression:** BDI **Anxiety:** BAI Initial substance use scale	**Any IPV:** 100% **Physical IPV (WEB):** 95%	**IPV-BI:** 95% (*n* = 38) Median BISA Score: 4 ± 2.12 **Non-IPV-BI:** 75%	**PTSD:** 95% **Depression:** 62% **Anxiety:** 58% **Substance use:** Max years 14 ± 9.88
McFadgion (2014) (65), United States *Thesis*	**Objective:** Explore the mechanisms, or underlying factors, of probable traumatic brain injuries among abused women. **Design:** A sequential mixed-methods design with quantitative survey data and qualitative semi-structured interviews.	Women who lived in domestic violence shelters or accessed services from domestic violence shelters in the District of Columbia metropolitan area. [*N* = 51]	**IPV in last 5 years:** CTS2 ^*^Physical abuse in last 5 years an inclusion criterion	**TBI and Strangulation** Brain Injury Screening Questionnaire and the CTS-I.	**PTSD:** PTSD Symptom Scale-Interview	100%	**Blows to the Head from Physical Abuse:** 79% (*n* = 37 of 48 providing responses) Average blows to the head 1.88 ± 1.19. 3+ times: 44% CTS scores (*n* = 47): 43.23 ± 47.48.	**PTSD (mean score):** 29.02 82% (*n* = 42) reported experiencing symptoms of traumatic stress. Women who experienced post traumatic stress symptoms were significantly likely to also experience a blow to the head from being physically abused (*r* = 0.459, *p* < 0.05)
Haag et al. (2022) (10), Canada	**Objective:** To explore the impact of the COVID-19 pandemic on survivors and service providers. **Design:** Qualitative, participatory approach using semistructured individual or group interviews	Overall: [*N* = 24] Survivors: [*n* = 6] ^*^Service providers and employers comprised the remaining sample	Women survivors of IPV were recruited through a Knowledge-to-Practice (K2P) network and with the assistance of frontline workers.	**TBI and Strangulation** Identification through questions about experiences indicative of BI.	Open-ended qualitative interview question about impacts of COVID-19 on health.	100% of survivors	Survivors: 100% All survivors interviewed had experienced hits or injury to the head, face, or neck, and all but one endorsed a resulting loss or alteration of consciousness, suggestive of BI.	Survivor reports of worsening stress, anxiety and depression from lack of social activity, fear of contracting the COVID-19 virus and financial instability. Increased isolation, loneliness, and fear were reported widely, as women were no longer able to access critical informal support networks.
Gabbe et al. (2022) (50), Australia	**Objective:** Compare the epidemiological profile, in-hospital and 6-month and 12-month patient-reported outcomes of major trauma patients w/ TBI injured through IPV and other interpersonal violence. **Design:** Population-based cohort study	Adult major trauma cases w/TBI in Victoria, Australia' trauma hospital system. [*N* = 1,062]	IPV and other interpersonal violence identified through the intent of injury variable and cross-checked against the text narrative of the injury event and the ICD-10-Australian Modification Chapter XX codes for consistency.	**TBI:** Defined as the presence of an Abbreviated Injury Scale diagnosis code for concussion, skull fracture, or intracranial injury as these codes include evidence of brain pathology or altered brain function. ^*^TBI an inclusion criterion	ICD-10-Australian Modification diagnosis codes mapped to indicator variables for preexisting mental health, drug, and alcohol conditions.	*n* = 52 (73% female). Annual incidence: 0.11/100,000 or 5.2 cases per-year	**TBI:** 100% of study population. 55% (*n* = 1,062 of 1,923) of adult major trauma cases due to interpersonal violence. 5% (*n* = 52 of 1,062) of interpersonal violence related TBI due to IPV	**Preexisting MHSU:** IPV: 32.0% (*n* = 16); Other Violence: 38.2% (*n* = 386). **No preexisiting MHSU:** IPV: 68.0% (*n* = 36); OV: 61.8% (*n* = 624)
Roberts and Kim (2005) (40), United States	**Objective:** Examine the link between chronic woman battering and head injuries. **Design:** Secondary analysis of interview data	Battered women who participated in a larger exploratory study [*N* = 52]	Chronically battered women based on larger study recruitment	**TBI** Reporting head or neck injuries when describing domestic violence related injuries ^*^Inclusion criterion	Not specified. PTSD, depression, and anxiety reported.	100%	**Head or Neck Injury:** 100% (32.5% or 52 of the 160 participants in larger study)	**PTSD symptoms**: All had frequent nightmares, 50/52 reported having flashbacks **Depressed mood:** 86% in depressed moods and/or taking medication for severe depression **Anxiety:** All 12% took medication for depression and panic attacks.
Likitlersuang et al. (2022) (67), United States	**Objective:** Identify the structural and functional impacts of IPV and IPV-related TBI on neural integrity **Design:** Pilot neuroimaging study	Community dwelling women survivors of IPV who screened positive for PTSD. [*N* = 45]	**Lifetime IPV:** Self report (1+ incidents of physical, psychological, and/or sexual violence) ^*^Inclusion criterion	**TBI** BAT-L/IPV	**PTSD:** CAPS-5 ^*^Positive screen on PCL-5 an inclusion criterion	100%	**Total TBI:** 51% (*n* = 23) **IPV-TBI:** 33% (*n* = 15) **Non-IPV TBI:** 18% (*n* = 8)	**PTSD (CAPS-5 mean score** **±** **SD)** TBI (*n* = 23): 37.0 ± 7.95 Control (*n* = 22): 30.0 ± 6.09 Significantly different (*p* = 0.002). IPV-TBI (*n* = 15): 38.1 ± 7.05 Non-IPV TBI (*n* = 8): 35.0 ± 9.58 No significant difference (*p* = 0.432)
Wong et al. (2020) (58), Hong Kong	**Objective:** (a) Identify the occurrence of mTBI in Chinese abused women admitted to emergency units; (b) compare the sociodemographic and past-year IPV characteristics between IPV abused women w/ and w/o mTBI; and (c) Examine the physical, mental, and cognitive functioning in abused women w/ mTBI, controlling for sociodemographic and IPV variables. **Design:** Cross sectional study	Chinese abused women at emergency units in four major local hospitals providing healthcare services to urban and rural catchment areas of Hong Kong between January 2014 and October 2016. [*N* = 86]	**Past-year IPV:** CTS-2 IPV ^*^Presenting injuries due to IPV an inclusion criterion	**mTBI** (a) ‘Have you ever lost consciousness or experience a period of being dazed and confused because of a head injury (within 30 min)?'; and (b) “Have you ever experienced memory dysfunction at the time of a head trauma?” ^*^Severe TBI [GCS < 13, LOC > 30 min, memory loss >24 h] excluded	**Anxiety and Depression:** Hospital Anxiety and Depression Scale (14-item Chinese version) **Health-related quality:** Chinese version of the 12-item Short Form Health Survey (physical and mental components)	100% Past-year psychological (but not physical or sexual) abuse significantly associated w/ IPV-related mTBI (*p* = 0.003).	**IPV-related mTBI**: 24.4% (*n* = 21) **IPV-related head injury subthreshold for mTBI:** 31.4% (*n* = 27) **Post-concussion symptoms:** (*n* = 85)	No reporting on the Hospital Anxiety and Depression Scale. Both the Physical and Mental Component Summary of quality of life were significantly different between abused women w/ and w/o mTBI (*p* = 0.04 and *p* < 0.0001, respectively). Mental Component Summary still significant after adjusting for past-year sociodemographic variables and IPV.
Brown et al. (2019) (47), United States	**Objective:** Examine the efficacy of neurofeedback in IPV survivors who experienced mTBI as measured by symptoms of disability, depression, anxiety, and PTSD, and changes in the brain. **Design:** Experimental pilot study using neurofeedback	Domestic violence program in Texas, individuals (31 females 1 male) experiencing IPV [*N* = 32]	Domestic violence program ^*^IPV an inclusion criterion	**TBI** qEEG data compared to TBI dataset	**Depression:** PHQ-9 **Anxiety:** Severity Measure for Generalized Anxiety Disorder **PTSD:** NSESSS	100%	Electrophysiological similarity to TBI: 69% (*n* = 20) (using qEEG data)	**Depression (mean score):** 16.44 “moderately severe depression” **Anxiety (mean score):** 2 “moderate anxiety” **PTSD (mean score):** 2.42 “moderate PTSD”
Marcantonis (2004) (64), United States *Thesis*	**Objective:** Examine the prevalence of TBI in a sample of women in battered women's shelters in New Jersey. **Design:** Between subjects design with quantitative methods.	Women in battered women's shelters and groups for survivors of domestic violence in New Jersey. [*N* = 29]	Recruited from battered women's shelters. ^*^Inclusion criterion	**TBI** TBI Questionnaire	**Depression:** BDI **Anxiety:** BAI **PTSD**: Penn PTSD	100%	**Any Cause TBI:** 25% (*n* = 7) **IPV-related TBI:** 21% (*n* = 6) 43% (*n* = 12) reported having sustained some insult to the head due to battery that resulted in feeling dazed and confused without LOC	**Depression [mean scores** **±** **SD; % (*****n*****)]** All (*n* = 23): 19 ± 13.19 IPV-TBI (*n* = 6): 83% (*n* = 5) **Anxiety [mean scores** **±** **SD; % (*****n*****)]** All (*n* = 28): 15 ± 13.34 IPV-TBI (*n* = 6): 83% (*n* = 5) **PTSD [mean scores** **±** **SD; % (*****n*****)]** All (*n* = 28): 27.28 ± 12.43 Statistically significant (*p* = 0.031) 36% (*n* = 10) met diagnostic criterion for PTSD IPV-TBI (*n* = 6): 66% (*n* = 4) ^*^Reported totals for individual scores varied from the overall sample size
Monahan and O'Leary (1999) (52), United States	**Objective:** Inquiry into the incidence, prevalence, and presenting symptomatology of head injury among battered women **Design:** Descriptive study using chart review	Battered women residing in a domestic violence shelter [*N* = 26]	Shelter for battered women ^*^Inclusion criterion	**TBI** Medical history form asking about head injury from partner or from childhood	Self-report and during councelling intake/followup—DSM-IV [substance abuse and depressed mood]	100%	**Head Trauma:** 35% (*n* = 9) **LOC:** 44% (*n* = 4)	**History of substance abuse:** ALL (*N* = 26): 31% (*n* = 8) Head-injured battered women (*n* = 9): 33% (*n* = 3) Non-head-injured battered women (*n* = 17): 29% (*n* = 5) **Depressed mood:** Head-injured battered women (*n* = 9): 44% (*n* = 4) Non-head-injured battered women (*n* = 17): 35% (*n* = 6)
Oden (2000) (57), United States, *Thesis*	**Objective:** Provide an exploratory analysis of the how often head injuries occur among battered women who seek help at battered women's agencies. “It is the intention of this study to focus on African American women to give them an opportunity to have their voices heard.” **Design:** Descriptive case-control study	African American women participating in programs for battered women throughout the Bay Area. [*N* = 64]	Recruited from battered women's shelters. ^*^Inclusion criterion	**TBI:** Semi-structured interview asking about fequency of blows to the head, occurrence of LOC and post-traumatic amnesia. ^*^Women with head injury from any cause other than IPV were excluded	**Various:** The Millon Clinical Multiaxial Inventory-III. ^*^Women with current and extreme history of alcohol or drug abuse, psychosis, or history of neurological conditions excluded	100%	**Hit in the Head:** 86% (*n* = 55) Average hits: 255 times (range 1–4,988). Average hits w/LOC: 6 (range 1–156)	50% of the women reported a psychiatric condition (52% depression, 26% combination anxiety and depression, 6% anxiety). **Anxiety (mean score):** Head injury (*n* = 51): 71.33; No head injury (*n* = 13): 49.08. Statistically significant (*p* < 0.01). **PTSD (mean score):** Head injury: 64.47; No head injury: 44.46. Statistically significant (*p* < 0.05). **Depression (mean score):** Head injury: 55.02; No head injury: 37.62. ^*^Depression scores reported as statistically significant (p < 0.10)

BAI, Beck Anxiety Index; BAT(-L), Boston Assessment of TBI(-Lifetime); BDI, Beck Depression Index; BI, brain injury; BISA, Brain Injury Severity Assessment; CAPS (-IV/5), Clinician Administered PTSD Scale (for DSM-IV/5); CAS, Composite Abuse Score; CESD, Center for Epidemiologic Studies Depression Scale; CTS, Conflict Tactics Scale; DASS, Depression Anxiety Stress Scale; DSM, Diagnostic and Statistical Manual of Mental Disorders; DOVE, Domestic Violence Home Visitation; GAD, generalized anxiety disorder; HARK, Humiliation/Afraid/Rape/Kick; HELP(S), Hit in the head, Emergency room treatment, Loss of consciousness, Problem because of hit to the head or strangulation, (other sickness they have experienced); ICD, International Classification of Diseases; IPV, intimate partner violence; LOC, loss of consciousness; MH, mental health; NSESSS, National Stressful Events Survey PTSD Short Scale; OSU-TBI-ID, Ohio State University TBI Identification Method; PCL, PTSD Checklist; PHQ, Patient Health Questionnaire; PTSD, post traumatic stress disorder; SCID-5, Structured Clinical Interview for DSM-5 Disorders; (m)TBI, (mild) traumatic brain injury; VA, Veterans Affairs; VHA, Veterans Health Administration; w/, with; w/o, without; WEB, Women's Experience of Battering.

^§^Articles reporting on the same study. ^*^Indicates a note or comment related to inclusion/exclusion criteria.

#### 3.1.1. Identifying intimate partner violence (IPV)

Experience of IPV was an inclusion criterion for almost all included studies, with one exception: Gabbe et al. (50) looked at rates of IPV among violence-related TBI, reporting an annual incidence of 0.11/100,000. Most studies identified survivors of IPV either through recruitment sites (e.g., women's shelters; 37%, *n* = 11) or *via* self-report of abusive relationships (24%, *n* = 7) with recruitment through a variety of sites. The remainder identified IPV using screening tools, with the most common being the Conflict Tactics Scale (CTS, *n* = 6) (43, 56, 58, 60, 65, 71). Additional tools included the Humiliate/Afraid/Rape/Kick tool (HARK tool, *n* = 2) (27, 59) and the Women's Experiences of Battering (*n* = 2) (55, 61).

While most studies included individuals who had experienced IPV at any point in their lifetime, there were several studies that required IPV within a certain timeframe. Four studies assessed for IPV within the last year (27, 51, 58, 60), Mittal et al. (55) required IPV within the last 3 months, and Saleem et al. (49) required an incident of physical IPV within the past 60 days. Two studies required participants to have presented to the recruitment site (emergency department and family advocacy center) with IPV-related injuries (48, 58).

#### 3.1.2. Assessing for brain injury (BI)

Identification of BI varied greatly across studies. In most instances, studies assessed for possible or probable BI by asking about instances of hits to the head, face, or neck followed by a period of altered or loss of consciousness, or by asking about instances of strangulation or choking. More than half of the included studies (*n* = 15) assessed for BI using screening tools or diagnostic interviews, with the Boston Assessment of TBI (62, 63, 67), Brain Injury Severity Assessment (61, 66, 68), and Veterans Affairs TBI screening tool (27, 59, 60) the most commonly used in three studies each. Gabbe et al. (50) used diagnostic codes to identify TBI in a health administrative dataset and Brown et al. (47) identified survivors with electrophysiological similarity to TBI *via* EEG. Three of the five studies focusing on strangulation simply asked if or how frequently survivors had been choked and/or strangled (51, 54, 55). The remainder of the studies assessed for BI using self-report that was not further specified. Some studies specifically excluded individuals with more severe BI (58, 66) or BI that was not IPV related (43, 49, 57, 66) and a subset of studies reported on BI from other causes in addition to IPV-BI (61, 62, 64, 66, 67).

As previously noted, most articles discussed BI broadly, including both TBI and strangulation (*n* = 15). Some focused on TBI specifically (*n* = 8), and others focused on strangulation (*n* = 5). Table 1 shows included articles stratified by these categories. Several studies in each category had BI as an inclusion criterion (40, 48, 50, 51, 53, 59, 69). Among studies where BI was not an inclusion criterion, the prevalence of strangulation ranged from 13 (56) to 93% (49), the prevalence of BI ranged from 19 (60) to 100% (68), and the prevalence of TBI ranged from 21 (64) to 69% (47). There was also a noted relationship between BI and IPV scores or experiences. Mittal et al. (55) reported significantly higher scores on two IPV scales among women who were strangled compared to women who were not, and Wong et al. (58) reported a significant association between past-year psychological (but not physical or sexual) abuse and IPV-related mild TBI.

#### 3.1.3. Assessing for mental health (MH) concerns

Of the 28 included articles, most either investigated anxiety, depression, and PTSD together (40, 43, 47, 57, 59, 61, 64, 66, 68) or MH as a broad concept (i.e., type of MH was not specified) (10, 48, 50, 51, 58, 69). Among the 17 articles measuring PTSD, the most commonly used measures were the Clinician administered PTSD scale (CAPS, *n* = 7) (43, 61–63, 66–68) and the Post-traumatic Stress Disorder Checklist (PCL, *n* = 5) (27, 59, 60, 62, 63), with two studies using the PCL to screen for PTSD and the CAPS to diagnose (62, 63). Assessment of both depression and anxiety was much more varied. Four of the 15 studies measuring depression used the Center of Epidemiologic Studies Depression Scale (55, 56, 59, 60). Three studies used the Beck Depression Inventory and Beck Anxiety Inventory to measure depression and anxiety, respectively (61, 64, 68), and two studies reported on anxiety and depression based on the HELPS tool, reporting “problems because of a hit to the head or due to strangulation” (49, 70). Though included in our search terms, only three studies reported substance use (49, 52, 61). Full reporting on measures used can be found in Table 1.

### 3.2. Experiences of BI and MH concerns in survivors of IPV

All studies in this review specifically discussed MH, BI, and IPV in relation to one another, allowing us to explore the intersectional impact of MH concerns and BI among IPV survivors. All but three of the 28 included articles reported on MH concerns among survivors of IPV with BI. Those three looked at rates of IPV among individuals with BI, reporting on MH prevalence within that subset (50); and rates of BI among survivors of IPV with positive screen and diagnosis of PTSD, respectively (62, 63).

Prevalence of MH among individuals who had experienced IPV and BI was reported in 13 studies, with depression ranging from 25 to 86% across nine studies (40, 49, 52, 54, 57, 61, 64, 68, 70), anxiety ranging from 32 to 100% across eight studies (40, 49, 54, 57, 61, 64, 68, 70), and PTSD ranging from 29 to 100% across six studies (27, 40, 61, 64, 65). Two studies reported the prevalence of MH as a broader category, ranging from 32 to 41% (48, 50). Finally, two studies reported the prevalence of substance use ranging from 28 to 33% (49, 61).

Relationships between BI and MH concerns among IPV survivors were also explored in 14 studies. Studies reported statistically significant differences in PTSD scores (27, 56, 57, 60, 64–67), depression scores (55, 60, 66), anxiety scores (57), or composite mental health scores (56, 58) among IPV survivors with BI compared to those without. Two studies compared MH scores with BI severity scores rather than grouping survivors with and without BI, both reporting significant positive associations between BI and MH scores (43, 47). Furthermore, the presence of BI was noted to negatively impact outcomes in PTSD treatment (63), and persistent BI symptoms were associated with lingering insomnia, depression, and physical health concerns (59). McFadgion (65) also reported that IPV survivors who experienced post-traumatic stress symptoms were more likely to experience a blow to the head from physical abuse. Two qualitative studies exploring experiences of BI among survivors of IPV noted MH concerns were often exacerbated following physical abuse (51) and negatively impacted by the COVID-19 pandemic (10). It was further noted that survivors often continued to experience MH concerns even after leaving the abusive relationship (51).

### 3.3. Healthcare use and access

Many studies identified implications for health and healthcare among IPV survivors with BI and MH; however, less than half investigated healthcare use or access. Seven studies used healthcare settings for some or all their participant recruitment (50, 54–56, 58, 60, 69), two of which also reported healthcare seeking (54, 69). An additional five studies reported the number of survivors who sought healthcare because of IPV (40, 51, 52, 64, 70). One article specifically compared health service use among IPV survivors with and without BI, reporting significantly higher Veterans Affairs healthcare use among women veteran IPV survivors with BI than those without (60). Studies reported 18–62% of survivors received care for an IPV-related injury at some point following the abuse (40, 51, 52, 54, 64, 69, 70). A qualitative exploration of care seeking identified fear of the abuser and a reluctance to discuss the experience of IPV as barriers (51).

## 4. Discussion

This review identified 28 trail-blazing articles discussing BI and MH concerns among survivors of IPV. Studies focused on cis gender women in relationships with men and were predominantly conducted in the US. Overall, the prevalence of BI (strangulation, TBI, or both) among IPV survivors ranged from 13 to 93%, which is in line with previous estimations (14). The prevalence of MH concerns among IPV-BI survivors, which has not previously been assessed in a review, ranged from 25 to 100%. Studies used a wide range of methods for identifying IPV, MH, and BI, including *via* recruitment settings, single self-report questions, validated questionnaires, and medical diagnoses. These differences are likely to contribute to the large ranges in prevalence seen across studies.

Many studies reported significant differences in MH scores between IPV survivors with BI compared to those without or significant correlations between BI and MH scores. Though only explored in two studies, BI was shown to negatively impact PTSD treatment outcomes and both physical and mental health. The prevalence of healthcare seeking was explored in a subset of studies, ranging from 18 to 62% among studies that did not recruit solely from healthcare sites. One of those studies reported higher care use among IPV survivors with BI than those without (60). Fear of the abuser and a reluctance to discuss the experience were noted as barriers to accessing care (51).

This review highlights the small but growing pool of foundational work on the intersection of MH concerns and BI among IPV survivors, underscoring the high prevalence of co-occurring MH and BI among IPV survivors and identifying opportunities for future exploration, including the investigation into the healthcare-related impacts of this intersection on survivors and the health system. The high rate of BI among survivors combined with the higher severity of MH associated with BI indicates this is a significant intersection for investigation not only for healthcare systems but also for community care systems and society at large. As much is still unknown about this intersection, four broad categories in need of further investigation are highlighted below.

### 4.1. Identification

Defining and screening for BI among IPV survivors is an ongoing debate in the IPV-BI literature (14, 48, 72). While loss of consciousness is a strong indicator of BI, more subtle alterations of consciousness (e.g., feeling dazed, confused, seeing stars) are also indicative of BI (56) but may not be captured depending on the way questions are worded and the survivor's memory of the incident. Given that almost all the included studies relied on survivor self-report, and the measures used varied from asking about specific injuries resulting in loss of consciousness to any injury resulting in the alteration of consciousness, it is likely that even when using BI screening measures, BIs are missed among IPV survivors. Several studies reported on BI both as identified *via* screening tools and as identified through questions about hits to the head with alterations in or loss of consciousness. In some cases, the prevalence using the latter method was double that of the former.

It is worth noting that the identification of IPV, particularly in healthcare settings, poses its own challenges. In addition to the challenges with IPV survivors not wanting or being able to seek treatment noted in the included literature, there are also challenges with the identification and disclosure of IPV when survivors do seek care. Medical professionals may be reluctant to broach the topic for reasons including lack of training or resources to do so or a belief that IPV is beyond their scope of practice. This, combined with a survivor's reluctance to disclose, can impede identification of IPV, impacting professionals' ability to adequately support survivors.

### 4.2. Sex and gender

The studies included in this review focused predominantly on cis gender women in relationships with men. However, there were inconsistencies throughout the included studies in referring to survivors as women, which aligns with the social construct of gender, and as females, which aligns with physiological sex. Similar inconsistencies were found in the reporting of partner sex and/or gender. As the experience of BI is influenced by both sex and gender (73–75), and IPV impacts individuals across the gender spectrum, there is an opportunity in this growing field to explore experiences of BI and MH concerns across IPV survivors of all genders and sexes.

### 4.3. Healthcare seeking

Included articles that explored healthcare predominantly focused on whether or not women sought medical help, rather than survivors' self-perceived health needs or how comorbid conditions shaped their care-seeking. The experience of care seeking, whether through medical or community routes, and perceived care needs is an opportunity for exploration. Several articles provided recommendations for healthcare providers in their discussions; the field would benefit from an investigation of how survivors experienced healthcare or other services that could further develop those recommendations. This call is echoed in the literature, identifying the triple intersection as needing more focus particularly because MH that is comorbid with BI, both in IPV survivors and the broader population, requires different considerations for care and treatment than MH alone, and vice versa (31, 76–78).

An additional consideration in the discussion of care-seeking is the healthcare and social context in which the study was conducted. Only five of the included articles were conducted outside of the US (10, 50, 58, 61, 68). While there are many aspects of the lived experience of BI, IPV, and MH that are universal, context also plays a role. Financial accessibility of healthcare has implications for care-seeking. More research in diverse contexts with different healthcare systems, including systems with universal healthcare, would support a more complete understanding of survivor needs and experiences. The one study reporting on healthcare use among IPV survivors with BI found increased Veterans Affairs healthcare use, which is funded for US veterans (60). Further investigation of the experiences of IPV-related BI and MH in contexts outside of the US will be critical for shaping the response to this “parallel pandemic” of COVID-19 and IPV, particularly when it comes to healthcare or service access and use. For example, in April 2020, the Government of Canada acknowledged IPV as a critical problem, exacerbated by COVID-19, and invested $207.5 million to support organizations addressing homelessness and women experiencing gender-based violence (79). Given the system-wide barriers and challenges identified with respect to the IPV-BI intersection overall (80), more research in this area could help target future investments to the areas with the greatest impact.

### 4.4. Intersectional representation

Throughout the literature included in this study, the intersection of other aspects of identity, such as race, ethnicity, ability, or immigration status, and the impacts of MH and BI among IPV survivors remains largely unexplored. Three studies focused on the experiences of specific ethnic groups (56–58) and one case report discussed the experiences of two refugees identifying as IPV survivors with BI (53); however, only one of the cases explored the triple intersection. A more thorough look into the impact of intersecting identities is needed as there are increased risks and differing care needs for these groups. For example, Indigenous women are at particularly high risk for IPV, reporting 2.5 times higher rates of violence and a higher rate of resultant injury (81), yet preliminary work in the IPV-BI intersection working with Indigenous groups in Canada (82) suggests that resources developed for urban settler populations are ineffective for many First Nations and Inuit communities for myriad reasons. A collaborative, Indigenous-led approach to developing culturally sensitive community-based resources and interventions is needed to support Indigenous survivors and their communities.

### 4.5. Strengths and limitations

This scoping review is the first, to the best of our knowledge, exploring the combined experience of IPV, BI, and MH. It provides insight into the prevalence of BI and MH among IPV survivors; identifies the wide variety of methods used to identify BI, MH, and IPV; and synthesizes the relationships between them as currently understood in the literature. The review used a comprehensive and purposefully broad search strategy across five databases that were not limited by date or language, maximizing the published literature captured in the initial search. The two-stage, systematic screening process, as well as the high proportion of agreement among reviewers, further minimized the risk of excluding relevant articles.

We acknowledge several limitations to this review. While the search was not limited by language, we were not able to review full-text articles in languages other than English, resulting in the exclusion of five articles at the full-text stage (3% of articles reviewed). In addition, any unpublished literature, reports, or briefs that may be present in gray literature were not captured in our search. Including gray literature has the noted benefit of providing a more comprehensive overview of available evidence on a subject; but, its inclusion poses a significant challenge in the increased human resources required to find, manage, and review these records (83). Unfortunately, due to resource limitations, a systematic search of the gray literature was not possible. Finally, we recognize that the findings presented are limited by the included literature, which predominantly used small (*N* < 50) convenience samples. It can be challenging to engage IPV survivors in research for a variety of reasons including willingness to disclose and safety concerns. Furthermore, many of the studies recruited through shelters and related domestic violence support centers, which impacts the type of individuals captured in the research.

### 4.6. Latest contributions

The literature focusing on the triple intersection is growing rapidly, as indicated by the majority of the included articles being published in the last 5 years. In the time between running our last database search and the publication of this review, we are aware of the publication of three additional articles reporting on the triple intersection. Chiou et al. (84) investigated depression severity in a subsample of IPV-BI survivors explored in another article included in this review (70), finding 64% of their participants endorsing moderate to severe depression based on the Beck Depression Inventory. Oakley et al. (85) assessed the willingness of IPV survivors to be screened for BI, with 88% of their sample screening positive for probable TBI on the HELPS tool. Among the sample with a positive TBI screen, 89 and 78% reported depression and anxiety, respectively. Finally, Quiroz Molinares et al. (86) explored BI among Colombian women survivors of IPV, finding 31% experienced BI and a significant correlation between BI score and depression when controlling for abuse severity and various socioeconomic factors. The findings from these articles align with those reported in the review.

## 5. Conclusion

This review highlighted the foundational and growing pool of literature on the triple intersection of IPV, BI, and MH and draws attention to the numerous opportunities for future work, such as increasing our understanding of BI and MH among IPV survivors, better representing the diversity of individuals experiencing IPV, and exploring service-related needs and experiences to inform policy and practice.

## Data availability statement

All data analyzed as part of this scoping review is from publicly available published literature.

## Author contributions

This study was conceptualized by DT, HLH, AC, and CMW in consultation with EN and VC. DT developed the search strategy, ran the database searches finalized analyses, and drafted the manuscript. DT, AM, EC, and SCG conducted screening and contributed to the analyses. All authors contributed to the manuscript revision, read, and approved the submitted version.
