# Supplementary material for: Exploring the intersection of brain injury and mental health in survivors of intimate partner violence: A scoping review
Source: Front Public Health. 2023 Mar 2;11:1100549. doi: 10.3389/fpubh.2023.1100549 (PMC10018197; doi:10.3389/fpubh.2023.1100549)
Supplement: Supplementary file 1 [file Data_Sheet_1.docx]

## Search Strategy

**Ovid MEDLINE: Epub Ahead of Print, In-Process & Other Non-Indexed Citations, Ovid MEDLINE® Daily and Ovid MEDLINE® (1946-Present)**

1. Domestic Violence/ or (domestic adj3 (abus* or violen*)).tw,kf.
2. exp Intimate Partner Violence/ or ((partner* or spous* or wife or wives) adj3 (abus* or violen*)).tw,kf.
3. Sex work/ or (sex work).tw,kf. or (sex adj3 industr*).tw,kf. or (prostitut*).tw,kf. or (sex* adj1 transact*).tw,kf.
4. exp Battered Women/ or ((batter*) adj3 (wife or wom* or wives)).tw,kf.
5. (intimate violence or interpersonal violence).tw,kf.
6. 1 or 2 or 3 or 4 or 5
7. exp Brain Injuries/ or traumatic brain inj*.tw,kf.
8. exp Brain Concussion/ or concuss*.tw,kf.
9. exp Head Injuries, Penetrating/ or exp Head Injuries, Closed/ or head inj*.tw,kf.
10. exp Post-Concussion Syndrome/ or post-concuss.tw,kf.
11. (strangle* or strangulat*).tw,kf.
12. exp Neck Injuries/ or exp Asphyxia/
13. exp Facial Injuries/ or face injur*.tw,kf.
14. 7 or 8 or 9 or 10 or 11 or 12 or 13
15. exp Mental Health/ or mental health.tw,kf.
16. exp Depressive Disorder/ or exp Depression/ or (depression? or depressed or depressiv* or MDD).tw,kf.
17. exp Stress Disorders, Post-Traumatic/ or PTSD.tw,kf. or post-traumatic stress disorder?.tw,kf.
18. exp Anxiety/ or exp Anxiety Disorders/ or anxiety.tw,kf.
19. exp Mood Disorders/ or mood disord*.tw,kf.
20. Substance-Related Disorders/
21. ((drug or substance? or alcohol or opioid? or amphetamine? or cocaine or marijuana or cannabis or phencyclidine or benzodiaz*) adj2 (misuse or abuse* or addict* or depend*)).tw,kf.
22. 15 or 16 or 17 or 18 or 19 or 20 or 21
23. 5 and 13 and 21
